# Ultra-processed foods and allergic symptoms among children and adults in the United States: A population-based analysis of NHANES 2005–2006

**DOI:** 10.3389/fpubh.2022.1038141

**Published:** 2022-11-03

**Authors:** Weiliang Kong, Yilian Xie, Jingjing Zhong, Chao Cao

**Affiliations:** Department of Respiratory and Critical Care Medicine, Ningbo First Hospital, Ningbo, China

**Keywords:** IgE, allergy, asthma, eczema, UPFs, NHANES

## Abstract

**Design:**

There is a strong correlation between dietary intake and allergic diseases. Ultra-processed foods (UPFs) are gradually becoming dominant worldwide and causing health problems for children and adults. We hope to determine whether links exist between UPFs and allergic symptoms.

**Methods:**

We investigated data from 2,736 children (16–19 years) and 4,256 adults (≥20 years) from the National Health and Nutritional Examination Survey (NHANES) 2005–2006. The associations between the mean UPFs contribution to total energy intake and all allergic symptoms (IgE, current asthma, allergy, rash, sneeze, wheeze, eczema, and hay fever) were estimated by weighted multivariate logistic regression.

**Results:**

Logistic regression analysis showed UFPs were negatively associated with IgE levels in children. Those with higher quartiles had a reduced risk from 16% (OR, 0.84, 95%CI, 0.55 to 1.28) to 34% (OR, 0.66, 95%CI, 0.49 to 0.89), p for trend = 0.006. UPFs were also positively related to current asthma in children with an increased risk of 11% (OR, 1.11, 95%CI, 0.79 to 1.56) to 76% (OR, 1.76, 95%CI, 1.10 to 2.82), p for trend = 0.0393. UPFs were also associated with eczema in girls. But there was no association observed between UPFs and allergic symptoms in adults.

**Conclusion:**

Our results suggested that UPFs assessed by the NOVA system were associated with IgE, current asthma in children, and eczema in girls. These results further support the need to test the association of modern dietary patterns with allergic symptoms.

## Introduction

Food allergies are a serious health problem (1), and they are commonly reported worldwide and continue to increase (2, 3). Data from the National Health and Nutrition Examination Survey (NHANES, 2007–2010) show the prevalence of food allergy was 6.5% in children and 10% in adults in the USA (4). While in the HealthNuts cohort, a study conducted in the Australian state of Victoria, there were unique insights into the accurate prevalence of IgE-mediated and oral food challenge-confirmed food allergies, which is 11.0% at age 1 year (5). A nonnegligible annual 5.7% increase in food allergy admissions was noticed in the UK (6). They can result in a wide range of combinations of signs and symptoms and can be involved in any organ system including the skin, the gastrointestinal tract, the respiratory system, and the cardiovascular (1). It is likely to be linked to an increasing preference for a more modern and western diet (7), which is characterized by a high intake of saturated fat, processed foods, pre-packaged foods, and added sugar in dairy products (8). Currently, ultra-processed foods (UPFs), as defined by the NOVA system, have already become a significant part of the western diet (9). They are industrial formulations typically composed of substances derived from additives and foods, including large amounts of added sugar, and usually require no cooking (9). The consumption of UPFs has increased rapidly among children and adults (10, 11). Epidemiological studies indicate that a high intake of UPFs is correlated with the development of several chronic diseases such as obesity (12, 13), insulin resistance (14), metabolic syndrome (15), dyslipidemia (16), hypertension (17), and cardiovascular disease (18). As one of the proxies for a low-quality diet, UPFs have gradually become an important concept in modern and western diets (19).

Substantial correlations have been established between unhealthy or pro-inflammatory diets and the pathogenesis of food allergy or asthma (20, 21). These include the association between a high intake of free fructose-containing beverages and allergy (20). Additionally, studies involving UPFs have suggested that biscuits, sweets or candies, processed meats, drinks, and packaged snacks enhance the risk of asthma and wheeze among Brazilian adolescents (22). However, there is also evidence to suggest the opposite. According to the data from the Pelotas birth cohort study, there were no significant associations found between UPFs consumption and asthma or wheeze during childhood or adolescence (23). To date, associations between UPFs and all sites of allergy-related symptoms have not been well demonstrated. In this article, we examine the relationship between UPFs and IgE and various allergic symptoms based on the data from the NHANES between 2005 and 2006.

## Methods

### Design

The national cohort study was conducted and utilized the data from 2005 to 2006 from the National Health and Nutrition Examination Survey (NHANES). It was designed as a large, stratified, multistage probability cluster sampling study by the National Center for Health Statistics (NCHS) and CDC to represent the US population.

**Figure 1:**
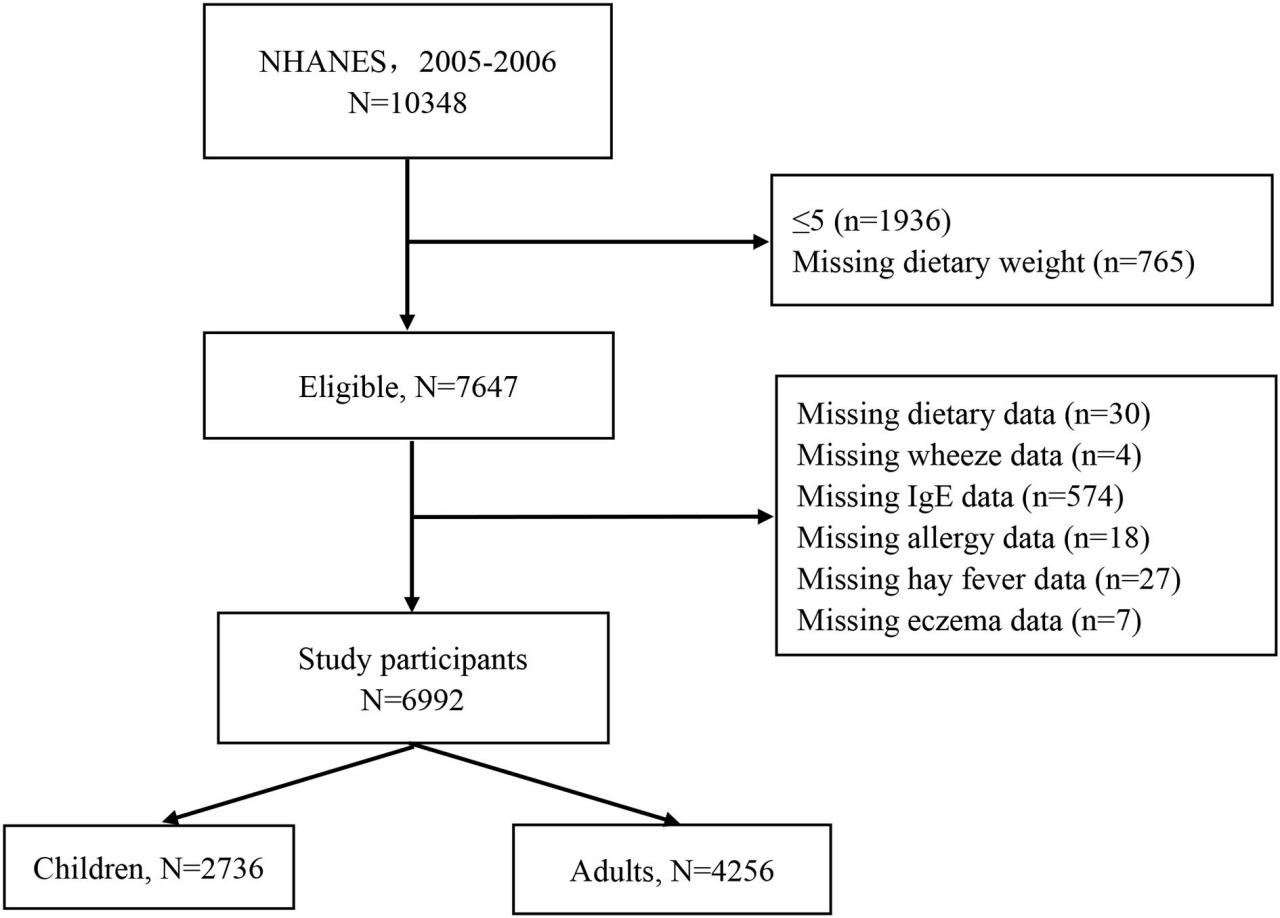
Participants flowchart.

During the 2005–2006 cycle, a total of 10,348 participants were interviewed. We first sequentially excluded 1,936 participants ≤5 years old and with missing dietary weight data (*n* = 765). Then, children and adults missing dietary data (*n* = 30), wheeze (*n* = 4), serum specific IgE (*n* = 574), allergy (*n* = 18), hay fever (*n* = 27), and eczema (*n* = 7) were excluded. Finally, 2,736 children and 4,256 adults were left for analysis (Figure 1).

### Assessment of UPFs

Trained interviews collected 24-h dietary recalls in-person following the U.S. Department of Agriculture (USDA) Automated Multiple-Pass Method, described in detail in the previous literature (24, 25). The 24-h dietary recall has been proven to be valid in previous studies (10, 11, 26). UPFs intake was assessed by administering two 24-h dietary recalls. All study samples (*n* = 9,169) had a first-day dietary recall by in-person household interview, and 90.1% (*n* = 8,264) had a second-day dietary recall by telephone interview. Nutrients were assigned to foods following the USDA Dietary Research Food and Nutrition Database (FNDDS) (27). The 8-digit food code was used to determine the food composition. Food and beverages consumed were recorded in grams, and this was then converted into calories by the FNDDS. UPFs intake was reported as the average intake to the total energy intake from two 24-h diet recalls according to the NOVA system.

NOVA has four food processing levels, namely, unprocessed foods, minimally processed foods, processed culinary in gradients' processed foods, and UPFs. All food items that matched the features of UPFs by the NOVA food classification system were listed and then applied to the FNDDS data (28). We pooled the energy of each individual's UPFs intake. A detailed description of UPFs and calorie estimation has been presented elsewhere (26, 29, 30). We referred to these studies to ensure the consistency and accuracy of food classification with previous studies. A more detailed description of the NOVA classification used in this analysis is shown in Supplementary Table S1.

### Assessments of allergic symptoms and sensitization

Allergy sensitization is indicated by a total IgE level of ≥150 kU/L. Self-reported allergic diseases were assessed using allergy, medical conditions, and respiratory health questionnaires during in-personal household interviews. We selected seven primary outcomes as this study's allergic symptoms, including hay fever, allergy, itchy rash, sneeze, wheeze, eczema, and current asthma. In some cases, allergic symptoms could be identified when participants provided both positive responses to questions such as “has a doctor told that you have hay fever/allergy/itchy rash/sneeze/wheeze/ eczema?” and “during the past 12 months, have you had an episode of hay fever/allergy/ itchy rash/ sneeze/ wheeze/ eczema?”. Current asthma was identified by two questions, both with positive responses to questions such as “has a doctor told that you have asthma?” and “do you still have asthma?”.

### Covariates

Potential confounding variables were chosen and adjusted in multivariate models. These included sociodemographic data, such as sex (male and female); age (categorized into 6–11 y, 12–19 y, 20–40 y, 40–59 y, and ≥60 y); race (non-Hispanic white, non-Hispanic black, Mexican, and others); family income ratio (<1 and ≥1); BMI [categorized into normal weight <25, 25 ≤ overweight<30, and 30 ≤ obesity by the World Health Organization (WHO) BMI classifications for adults and normal weight (<28.97 kg/m^2^), overweight (28.97 to <34.57 kg/m^2^), and obesity (≥34.57 kg/m^2^) for children according to the age- and gender-specific criteria by a previous study (31)]; and smoke status (categorized into the current smoker, former smoker, and never smoker by self-reported questionnaire for adults). Tobacco exposure among children was assessed and categorized by serum cotinine (32), active smoker (cotinine ≥ 10 ng/ml), second-hand smoker (0.015 ng/ml ≤ cotinine<10 ng/ml), and non-smoker (cotinine < 0.015 ng/ml). The housing characteristics were the presence of mildew smell (yes or no), furry animals (yes or no), and cockroaches (yes or no) in the home.

### Statistical analysis

To reflect the characteristics of the NHANES multiple sampling survey (33), data analysis was performed by the R (version 4.2.0, “survey” package). We used a Taylor-series linearization approach to express mean ± standard errors (SEs) for all continuous variables and mean (95%CI) for all categorical variables following the official guidance of NHANES. Then, the data were compared by Student's *t*-test and Cochran–Mantel–Haenszel Chi-square test. We then used multiple logistic regression models to estimate the odds ratio (OR) for all sites of allergy sensitization and various allergic symptoms comparing quartiles 2, 3, and 4 (a higher intake of UPFs) with quartile 1 of UPFs (%Kcal) among children and adults, respectively. P for trend was calculated using the median value of each quartile as a continuous variable in each model. Stratified analysis was conducted to further explore the consistency of UPFs consumption's effects on allergies in the relevant confounding groups, such as age, gender, and race/ethnicity. Finally, interaction analysis was conducted between UPFs and covariates by including the interaction terms in the multiple logistic regression.

## Results

### Characteristics of the study participants

The participant characteristics by quartile of daily UPFs energy intake are shown in Table 1. The study population comprised 2,734 children (6–19 years) and 4,257 adults (≥ 20 years). The mean ± SE values of UPFs were 62.1 ± 0.9% for children and 53.3 ± 0.6% for adults. The participants with a higher intake of UPFs were likely to be non-Hispanic black in adults but were less likely to be Mexican American in both children and adults. They were also more likely to be the younger (<60 years) and less likely to be the elder (≥60 years) adults. The participants with a higher intake of UPFs consumption had a lower prevalence of IgE-sensitization in children, from 26.64 to 21.41%, p _trend_ = 0.0162. However, they had a higher prevalence of allergy in children, from 18.22 to 28.46%, p _trend_ = 0.0452, and a higher prevalence of current asthma in children, from 17.30 to 33.31%, p _trend_ = 0.0194. They also had a higher prevalence of wheeze in adults, from 20.16 to 28.31%, p _trend_ = 0.0326.

**Table 1:** Characteristics of the study participants according to the quartiles of UPFs (%Kcal) among children and adults.

**Characteristics**	**by quartiles of UPFs(%Kcal) in children**					**by quartiles of UPFs(%Kcal) in adults**				
	**Q1**	**Q2**	**Q3**	**Q4**	**P trend**	**Q1**	**Q2**	**Q3**	**Q4**	**P trend**
Energy	2016.54 (71.27)	2196.47 (55.31)	2337.41 (93.99)	2241.69 (66.26)	0.0044	2120.66 (40.28)	2139.39 (42.36)	2225.55 (38.56)	2304.95 (56.16)	0.0005
**Sex**					0.9444					0.743
Female	23.45(2.09)	26.91(1.73)	24.30(1.61)	25.34(1.98)		23.00(1.06)	25.60(1.51)	25.54(1.19)	25.86(1.31)	
Male	21.21(1.85)	28.31(1.53)	27.95(1.89)	22.53(1.65)		25.23(1.88)	23.44(1.04)	25.02(1.83)	26.30(0.93)	
**Age**					0.3717					<0.0001
6–11	21.36(2.56)	31.82(2.47)	25.63(1.61)	21.19(1.96)		NA	NA	NA	NA	
12–19	22.93(2.76)	24.72(1.47)	26.60(2.30)	25.75(2.52)		NA	NA	NA	NA	
20–39	NA	NA	NA	NA		20.16(1.72)	22.91(1.24)	25.89(1.89)	31.04(1.24)	
40–59	NA	NA	NA	NA		23.62(1.59)	24.48(1.32)	25.17(1.29)	26.73(1.37)	
≥ 60	NA	NA	NA	NA		30.66(1.77)	27.21(1.88)	24.57(0.74)	17.56(1.37)	
**Race/Ethnicity**					0.3877					0.0033
Non-Hispanic white	20.06(2.37)	26.72(1.76)	28.77(1.76)	24.44(2.12)		22.89(1.39)	24.72(0.86)	26.59(1.49)	25.80(0.72)	
Non-Hispanic black	23.77(2.04)	27.44(2.13)	22.18(2.12)	26.61(2.47)		16.35(1.35)	22.05(1.28)	25.17(1.51)	36.44(1.90)	
Mexican	33.18(2.89)	30.04(2.87)	22.07(2.55)	14.71(2.31)		33.25(2.24)	28.68(2.79)	20.74(1.43)	17.33(1.68)	
Others	19.40(3.12)	30.36(5.28)	21.94(3.73)	28.30(4.76)		37.44(2.80)	22.46(2.98)	17.52(1.93)	22.57(3.05)	
**FIR**					0.2065					0.0414
<1	30.22(3.06)	27.28(2.23)	21.74(2.54)	20.76(3.18)		28.57(3.17)	25.36(2.04)	25.00(1.81)	21.07(1.61)	
≥1	20.31(1.96)	27.60(1.66)	27.08(1.82)	25.02(1.94)		23.46(1.18)	24.69(0.82)	25.31(1.17)	26.54(0.67)	
**BMI**					0.6317					0.1189
Normal	22.39(1.72)	28.59(1.42)	25.64(1.50)	23.37(1.55)		24.00(2.04)	25.79(1.18)	26.04(2.03)	24.17(1.26)	
Obesity	22.13(5.38)	22.42(4.58)	31.12(10.22)	24.33(9.87)		23.69(1.33)	23.69(1.01)	22.83(1.16)	29.80(1.29)	
Overweight	21.28(3.66)	20.08(4.26)	30.47(5.21)	28.16(5.52)		24.80(2.06)	24.52(1.53)	26.25(1.73)	24.43(1.57)	
**Tobacco exposure**					0.231					0.1421
No exposure	17.01(2.23)	29.46(2.76)	27.91(4.13)	25.62(3.57)		26.07(2.39)	26.41(2.49)	23.32(2.37)	24.20(2.88)	
Secondhand smoke	23.21(1.68)	28.46(1.39)	25.27(1.58)	23.07(1.39)		24.10(1.77)	24.65(1.32)	25.46(1.53)	25.78(1.31)	
Active smoker	24.81(4.69)	18.19(3.59)	28.05(4.42)	28.95(5.64)		23.01(2.39)	23.19(1.89)	26.02(2.18)	27.78(1.42)	
**Smoke Status**										0.5208
Never	NA	NA	NA	NA		22.97(1.60)	23.25(1.37)	26.07(1.05)	27.72(1.47)	
Former	NA	NA	NA	NA		26.77(1.55)	27.88(1.89)	23.58(1.73)	21.77(1.87)	
Active	NA	NA	NA	NA		23.57(2.08)	23.98(1.70)	25.42(2.10)	27.02(1.34)	
Animals	21.62(5.24)	31.89(6.56)	26.66(7.98)	19.83(4.59)	0.5238	17.14(5.22)	26.15(5.20)	25.13(5.24)	31.59(4.18)	0.1416
Cockroaches	28.24(3.39)	27.65(2.41)	23.59(4.66)	20.52(2.41)	0.0785	28.31(1.50)	21.58(2.09)	25.95(3.65)	24.16(2.34)	0.1849
Mildew	24.12(2.91)	26.51(3.47)	23.05(4.27)	26.32(4.16)	0.9465	20.69(2.19)	24.95(2.57)	26.87(2.43)	27.49(1.67)	0.1433
IgE	26.64(3.55)	28.50(2.84)	23.45(2.63)	21.41(2.41)	0.0162	25.60(2.35)	25.35(2.30)	22.35(2.12)	26.70(1.95)	0.5597
Allergy	18.22(2.07)	25.54(3.50)	27.78(3.35)	28.46(3.35)	0.0452	21.46(1.94)	22.31(2.26)	28.11(2.03)	28.13(2.23)	0.1211
Current asthma	17.30(2.40)	24.59(3.93)	24.80(4.82)	33.31(3.73)	0.0194	18.54(2.44)	22.22(2.75)	30.28(2.56)	28.96(3.75)	0.0497
Wheeze	21.40(2.73)	27.73(3.46)	24.49(4.20)	26.38(3.53)	0.6914	20.16(2.55)	22.34(2.44)	29.19(2.47)	28.31(2.39)	0.0326
Rash	19.97(4.30)	34.07(5.87)	27.53(5.16)	18.44(4.02)	0.3797	18.82(3.17)	23.44(2.94)	28.01(3.03)	29.73(3.34)	0.1291
Sneeze	21.60(3.41)	27.37(3.01)	21.55(2.15)	29.48(2.76)	0.2178	22.27(1.69)	24.31(1.21)	24.46(1.63)	28.96(1.52)	0.0957
Hay fever	15.81(4.56)	36.13(6.27)	20.37(6.72)	27.69(7.22)	0.522	29.45(3.50)	22.39(3.09)	23.70(2.54)	24.46(2.53)	0.2104
Eczema	16.74(3.51)	26.51(3.91)	29.92(3.54)	26.82(3.75)	0.1353	20.46(3.72)	29.51(3.77)	27.74(4.06)	22.28(2.88)	0.795

Weighted mean ± Se and Student's *t*-test for continuous variables.

Weighted %, mean (95% CI), and Cochran–Mantel–Haenszel Chi-square test for categorical variables. Trends across each quartile of UPFs consumption were assessed by *t*-test.

FIR, family income ratio.

### Association of UPFs with allergic symptoms

After conducting the multiple logistic regression analysis, the associations of consumption of UPFs with all sites of allergy-related outcomes among children and adults are shown in Table 2. We found negative and significant associations between UPFs and IgE sensitization among children in all three models. In the fully adjusted models (Model 3), when compared with the lowest quartile, participants with higher quartiles (Q2–Q4) had a decrease in the risk of IgE-sensitization event from 16% (Q2, OR, 0.84, 95%CI, 0.55 to 1.28) to 34% (Q4, OR, 0.66, 95%CI, 0.49 to 0.89), p for trend = 0.006. Additionally, all three models found significant and positive associations with current asthma. The ORs (95% CIs) across the increasing quartiles in Model 3 were 1.11 (0.79, 1.56), 1.12 (0.70, 1.80), and 1.76 (1.10, 2.82) compared with Q1, p for trend = 0.0393. However, we did not find any significant association between UPFs and allergy-related outcomes among adults.

**Table 2:** Odds ratios of the associations between UPFs and allergic symptoms in children and adults, NHANES (2005–2006).

**Allergic symptoms**	**Children**						**Adults**					
	**Model 1**	**p _trend_**	**Model 2**	**p _trend_**	**Model 3**	**p _trend_**	**Model 1**	**p _trend_**	**Model 2**	**p _trend_**	**Model 3**	**p _trend_**
**IgE**		0.0147		0.0295		0.0062		0.5529		0.6329		0.7133
Q1	ref = 1.00						ref = 1.00					
Q2	0.82(0.57,1.18)		0.85(0.57,1.27)		0.84(0.55,1.28)		0.96(0.69,1.34)		1.00(0.72,1.40)		1.07(0.75,1.52)	
Q3	0.68(0.43,1.08)		0.72(0.45,1.17)		0.68(0.42,1.12)		0.80(0.55,1.16)		0.83(0.57,1.22)		0.89(0.59,1.35)	
Q4	0.68(0.50,0.93)		0.69(0.51,0.95)		0.66(0.49,0.89)		0.95(0.72,1.26)		0.96(0.71,1.31)		0.98(0.68,1.40)	
**Allergy**		0.0437		0.1019		0.0797		0.1344		0.2476		0.2477
Q1	ref = 1.00						ref = 1.00					
Q2	1.14(0.77,1.69)		1.13(0.75,1.69)		1.09(0.71,1.69)		1.02(0.74,1.41)		0.98(0.71,1.37)		0.99(0.70,1.39)	
Q3	1.35(0.90,2.03)		1.29(0.83,2.02)		1.27(0.78,2.06)		1.33(1.04,1.69)		1.25(0.98,1.61)		1.28(1.01,1.62)	
Q4	1.56(1.03,2.35)		1.49(0.95,2.32)		1.46(0.96,2.23)		1.26(0.87,1.82)		1.19(0.82,1.72)		1.17(0.80,1.72)	
**Current asthma**		0.0175		0.0317		0.0393		0.0556		0.1181		0.1665
Q1	ref = 1.00						ref = 1.00					
Q2	1.16(0.81,1.67)		1.16(0.82,1.63)		1.11(0.79, 1.56)		1.19(0.92,1.54)		1.15(0.88,1.51)		1.17(0.90,1.54)	
Q3	1.25(0.74,2.10)		1.21(0.73,2.00)		1.12(0.70, 1.80)		1.61(1.20,2.17)		1.56(1.12,2.16)		1.54(1.10,2.17)	
Q4	1.96(1.24,3.08)		1.87(1.19,2.95)		1.76(1.10, 2.82)		1.48(0.99,2.23)		1.40(0.91,2.15)		1.38(0.86,2.22)	
**Eczema**		0.1407		0.2003		0.2607		0.792		0.4103		0.8288
Q1	ref = 1.00						ref = 1.00					
Q2	1.31(0.68,2.51)		1.22(0.61,2.41)		1.15(0.55,2.44)		1.46(0.82,2.57)		1.37(0.79,2.39)		1.53(0.84,2.79)	
Q3	1.60(0.94,2.71)		1.52(0.87,2.65)		1.54(0.88,2.69)		1.32(0.83,2.09)		1.19(0.73,1.96)		1.30(0.80,2.13)	
Q4	1.57(0.82,2.99)		1.48(0.76,2.87)		1.33(0.69,2.56)		1.01(0.64,1.58)		0.91(0.58,1.42)		1.05(0.63,1.73)	
**Hay fever**		0.4822		0.614		0.8363		0.2198		0.1772		0.1653
Q1	ref = 1.00						ref = 1.00					
Q2	1.87(0.77,4.57)		1.90(0.78, 4.66)		1.86(0.79, 4.34)		0.73(0.48,1.09)		0.69(0.46,1.03)		0.70(0.45, 1.08)	
Q3	1.10(0.44,2.73)		1.09(0.43, 2.74)		1.06(0.43, 2.62)		0.75(0.48,1.17)		0.68(0.43,1.09)		0.62(0.39, 0.97)	
Q4	1.65(0.66,4.15)		1.55(0.62, 3.90)		1.37(0.56, 3.35)		0.75(0.52,1.08)		0.70(0.46,1.05)		0.72(0.47, 1.10)	
**Rash**		0.4231		0.5524		0.6024		0.1009		0.0872		0.1071
Q1	ref = 1.00						ref = 1.00					
Q2	1.40(0.77,2.55)		1.44(0.80,2.58)		1.52(0.78, 2.96)		1.24(0.76,2.02)		1.28(0.77,2.13)		1.26(0.71,2.22)	
Q3	1.18(0.68,2.07)		1.25(0.71,2.20)		1.38(0.78, 2.44)		1.46(0.92,2.30)		1.51(0.96,2.37)		1.52(0.94,2.44)	
Q4	0.86(0.47,1.56)		0.88(0.48,1.63)		0.88(0.42, 1.85)		1.50(0.91,2.47)		1.58(0.95,2.63)		1.47(0.88,2.46)	
**Sneeze**		0.208		0.3582		0.3872		0.0899		0.1342		0.0985
Q1	ref = 1.00											
Q2	1.03(0.62,1.70)		1.02(0.62,1.68)		1.04(0.61,1.79)		1.11(0.87,1.41)		1.08(0.85,1.37)		1.09(0.85,1.40)	
Q3	0.81(0.49,1.33)		0.77(0.45,1.31)		0.79(0.44,1.43)		1.07(0.82,1.40)		1.03(0.79,1.34)		1.06(0.80,1.40)	
Q4	1.41(0.96,2.06)		1.33(0.90,1.95)		1.31(0.86,2.01)		1.33(0.99,1.79)		1.30(0.97,1.74)		1.30(0.98,1.74)	
**Wheeze**		0.6782		0.7729		0.9468		0.0312		0.0517		0.0658
Q1	ref = 1.00						ref = 1.00					
Q2	1.05(0.72,1.53)		1.04(0.71,1.52)		1.10(0.76, 1.58)		1.10(0.76,1.60)		1.10(0.76,1.59)		1.16(0.77,1.75)	
Q3	0.97(0.61,1.54)		0.95(0.58,1.55)		0.87(0.53, 1.43)		1.46(0.99,2.16)		1.45(0.98,2.13)		1.53(1.02,2.31)	
Q4	1.17(0.62,2.21)		1.13(0.59,2.17)		1.09(0.58, 2.05)		1.36(1.00,1.84)		1.35(1.00,1.82)		1.37(0.94,1.99)	

Model 1 adjusted for none.

Model 2 adjusted for gender, age, and race/ethnicity.

Model 3 adjusted for gender, age, race/ethnicity, family income ratio, BMI (categorical variables), smoke status (self-reported in adults), tobacco exposure (defined by cotinine), animals, cockroaches, and mildew.

P _trend_ was calculated by using the median value of each quartile as a continuous variable in each model.

Ref, reference.

In addition, when using IgE as a continuous variable (Supplementary Table S2), the multiple linear regression also showed there is a significant association among children with β coefficient (95% CIs), −17.46 (−90.42, 55.50), −68.75 (−116.33, −21.16), and −56.12 (−115.09, 2.86), p for trend = 0.02.

### Subgroup and interaction analyzes

Subgroup analyzes were performed by sex, age, and race/ethnicity (white, the others) among children and adults respectively (Table 3 and Supplementary Table S3). A significant interaction was observed between UPFs and the prevalence of eczema in children when stratified by sex (*p* = 0.02). We, thus, repeated the multiple logistic regression analysis in children after stratification by sex (Table 4). In this analysis, girls with higher UPFs consumption (Q2–Q4) had significantly increased odds of eczema (p for trend = 0.0114). In contrast, no significant association was found between UPFs and eczema among male children.

**Table 3:** The association between UPFs and allergic symptoms, stratified by selected subgroups in children.

			**IgE**	**Allergy**	**Current asthma**	**Eczema**	**Hay fever**	**Rash**	**Sneeze**	**Wheeze**
Age	6–11	Q1	ref = 1.00	ref = 1.00	ref = 1.00	ref = 1.00	ref = 1.00	ref = 1.00	ref = 1.00	ref = 1.00
		Q2	1.05(0.38, 2.86)	1.33(0.49,3.62)	2.02(0.94, 4.36)	1.23(0.51, 2.98)	3.91(0.82, 18.59)	1.34(0.49, 3.66)	1.18(0.50, 2.82)	1.13(0.56, 2.30)
		Q3	0.50(0.20, 1.26)	0.95(0.31,2.88)	1.59(0.49, 5.15)	2.57(1.15, 5.75)	1.61(0.15, 17.20)	2.01(0.75, 5.39)	0.79(0.28, 2.24)	0.93(0.48, 1.81)
		Q4	0.94(0.49, 1.79)	2.69(1.28,5.66)	2.12(0.78, 5.75)	1.13(0.48, 2.70)	2.50(0.39, 15.94)	0.56(0.15, 2.14)	1.46(0.69, 3.09)	1.38(0.60, 3.17)
	12–19	Q2	0.77(0.56,1.07)	0.93(0.50,1.75)	0.69(0.36, 1.33)	1.08(0.48, 2.44)	1.26(0.37, 4.25)	1.67(0.76, 3.68)	0.94(0.51,1.72)	1.10(0.59, 2.05)
		Q3	0.80(0.50,1.28)	1.46(0.74,2.90)	0.93(0.51, 1.69)	0.84(0.39, 1.80)	0.73(0.25, 2.15)	0.96(0.36, 2.59)	0.78(0.44,1.38)	0.85(0.34, 2.12)
		Q4	0.59(0.45,0.78)	1.00(0.50,2.00)	1.64(0.89, 3.02)	1.41(0.53, 3.76)	0.93(0.29, 3.03)	1.11(0.61, 2.04)	1.24(0.80,1.92)	0.99(0.37, 2.62)
	p for interaction		0.65	0.14	0.8	0.65	0.26	0.97	0.76	0.71
Sex	female	Q1	ref = 1.00	ref = 1.00	ref = 1.00	ref = 1.00	ref = 1.00	ref = 1.00	ref = 1.00	ref = 1.00
		Q2	1.11(0.61, 2.01)	1.44(1.11,1.87)	1.20(0.68, 2.14)	2.46(0.80, 7.58)	4.64(2.02, 10.65)	1.29(0.58, 2.89)	1.06(0.57, 1.99)	0.88(0.41, 1.89)
		Q3	0.71(0.42, 1.19)	1.85(1.10,3.14)	2.01(0.85, 4.75)	4.40(1.87,10.33)	2.17(0.49, 9.53)	1.60(0.83, 3.09)	0.88(0.45, 1.73)	1.54(0.63, 3.77)
		Q4	0.65(0.36, 1.20)	1.87(1.03,3.41)	1.55(0.71, 3.41)	2.88(1.05, 7.95)	2.58(0.79, 8.39)	0.66(0.28, 1.57)	1.39(0.89, 2.15)	1.31(0.58, 2.97)
	male	Q2	0.66(0.35,1.26)	0.77(0.36,1.64)	1.03(0.59, 1.79)	0.66(0.28, 1.55)	0.47(0.11, 2.06)	1.76(0.54, 5.69)	0.98(0.49, 1.96)	1.17(0.70, 1.95)
		Q3	0.68(0.36,1.27)	0.81(0.40,1.64)	0.60(0.29, 1.22)	0.64(0.29, 1.40)	0.45(0.14, 1.49)	0.85(0.27, 2.66)	0.72(0.33, 1.59)	0.50(0.21, 1.19)
		Q4	0.68(0.38,1.22)	1.09(0.59,2.02)	2.04(1.08, 3.83)	0.72(0.29, 1.82)	0.65(0.14, 2.96)	1.04(0.35, 3.10)	1.27(0.61, 2.63)	0.91(0.44, 1.88)
	p for interaction		0.89	0.16	1.0	0.02	0.16	0.93	0.38	0.23
Race/	White	Q1	ref = 1.00	ref = 1.00	ref = 1.00	ref = 1.00	ref = 1.00	ref = 1.00	ref = 1.00	ref = 1.00
Ethnicity		Q2	0.57(0.34,0.96)	0.93(0.52, 1.66)	1.21(0.64, 2.29)	1.09(0.42, 2.85)	2.79(1.05, 7.38)	2.11(0.60, 7.49)	1.04(0.51, 2.15)	1.41(0.62, 3.18)
		Q3	0.65(0.28,1.49)	1.15(0.54, 2.43)	1.27(0.52, 3.08)	1.24(0.64, 2.40)	1.26(0.40, 3.98)	1.67(0.58, 4.85)	0.68(0.30, 1.55)	1.09(0.40, 2.95)
		Q4	0.68(0.44,1.05)	1.28(0.75, 2.19)	2.08(0.99, 4.37)	1.08(0.50, 2.36)	1.03(0.30, 3.49)	0.89(0.25, 3.20)	1.15(0.63, 2.09)	1.17(0.36, 3.84)
	the others	Q2	1.25(0.81, 1.92)	1.44(0.73,2.81)	0.94(0.58, 1.52)	1.36(0.61,3.03)	0.54(0.13, 2.31)	1.06(0.47, 2.36)	0.96(0.58,1.60)	0.74(0.44, 1.26)
		Q3	0.78(0.50, 1.21)	1.63(0.91,2.94)	0.89(0.43, 1.86)	2.35(1.05,5.29)	0.75(0.17, 3.40)	1.06(0.69, 1.64)	1.11(0.67,1.83)	0.60(0.36, 1.00)
		Q4	0.70(0.45, 1.11)	1.91(1.02,3.60)	1.33(0.72, 2.46)	2.32(0.98,5.47)	2.27(0.50, 10.26)	0.86(0.42, 1.79)	1.70(1.26,2.28)	1.01(0.66, 1.55)
	p for interaction		0.67	0.35	0.32	0.28	0.14	0.98	0.28	0.8

All models were adjusted for gender, age, race/ethnicity, family income ratio, BMI (categorical variables), smoke status (self-reported in adults), tobacco exposure (defined by cotinine), animals, cockroaches, and mildew except the subgroup variable.

**Table 4:** The associations between UPFs and eczema by sex in children, NHANES 2005–2006.

**UPFs**	**Eczema in female**	**Eczema in male**
Q1	ref = 1.00	ref = 1.00
Q2	2.39 (0.70, 8.15)	0.60 (0.24, 1.50)
Q3	4.81 (1.87, 12.38)	0.55 (0.24, 1.27)
Q4	2.90 (0.97, 8.74)	0.67 (0.26, 1.71)
	p for trend = 0.0114	p for trend =0.4107

All models were adjusted for age, race/ethnicity, family income ratio, BMI, tobacco exposure, animals, cockroaches, and mildew.

## Discussion

In this nationwide population-based cross-sectional study, UPFs were significantly and negatively associated with IgE sensitization and were positively related to the prevalence of current asthma in children. Although no significant association was found for eczema, further stratified and interaction analyzes showed that the association was significant in girls.

Ultra-processed foods are not considered “real food” but affect human health by complex mechanisms involving the synergic effects of additives and nutrients lacking in them (34). However, information regarding their role in the allergic immune response is limited. IgE-mediated (allergic) food reaction is one of the major mechanisms of allergic reactions (35). The reaction is characterized by adverse immunological responses to specific food proteins. Considering the results from previous studies, higher levels of total IgE at baseline in participants were associated with increased intake of food proteins, such as egg, milk, peanut, and tree nuts (36). Because of the characteristics of UPFs (37), in patients with a higher intake of UPFs, it is difficult for the body to produce an allergic reaction (38, 39). On the other hand, it was reported that IgE sensitization and food hypersensitivity declined with increasing age (40). Our study found that IgE association was present in adults.

Allergic asthma is increasing, and diet changes represent a key factor in the increasing allergies (41). Fast food, processed food, and processed meat consumption in childhood have been proven to be associated with asthma development (42). Vitamin A, vitamin D, trace elements, and fiber could play a defensive role in airway immune reactions (43). A large retrospective study of 109,104 Brazilian adolescents [Brazilian study (22)] found positive associations between UPFs consumption and asthma. Another study of 971 adults reported that a high intake of processed meat causes worse asthma symptoms (44). Studies have provided positive associations between excessive fructose and the development of asthma among children (45, 46). These results expand on the knowledge about the relationship between UPFs and asthma. Our study also provided such a significant association. However, a crucial assumption underlying this analysis is that participants' exposure to UPFs is unlikely to vary heavily over the past year. Thus, we need to study more prospectively to reveal their association.

Eczema is a common chronic inflammatory skin disease in children (41, 47). Based on the data from the Isle of Wight Birth Cohort, the previous result suggested that men and women may experience various courses of eczema (48). The developmental trajectories indicated that the prevalence of eczema is equal to or slightly higher in men during adolescence but predominates in women during post-puberty (48). Our finding of a positive association between UPFs and eczema in girls suggests that girls with eczema are more susceptible to such low-quality diets. Besides, it is noticed that the dose–response trend declined in the highest quartile which suggests a potential threshold effect.

The possible and major mechanisms of food-induced hypersensitivity reactions include dual allergen exposure, the vitamin D hypothesis, and the hygiene hypothesis (35). A high intake of UPFs represents a high intake of processed foods, saturated fat, and sugar, but fewer proteins (allergens) (10, 11). It may contribute to decreased immediate-related hypersensitivity reactions (22). However, this does favor the formation of allergic immune defense in childhood (43). Previous studies have shown a reduction in allergic symptoms by repeated exposures to low doses of the allergen in children (49, 50). Recently, the Learning Early About Peanut Allergy (LEAP) study has validated that the onset of peanut allergy declines after low- and moderate-risk children (4–11 months of age) are exposed to peanuts at an early stage (51, 52). It has also shown that the effect is long-lasting after 1 year of continuing avoidance (53). In addition, available evidence has shown that the gut microbiome is always implicated in the development of food allergies (54, 55). Commensal microbiota can modulate immune development and the formation of a healthy immune response to food (56). On the other hand, various diets influence intestinal permeability and gut microbiome (57, 58). Some observational studies have provided insight into the association between vitamin D deficiency and food allergies in children (59). The insufficiency of vitamin D is identified frequently in multiple food allergies compared with children with single food allergies (60). At the same time, vitamins and trace elements, including vitamin D, are impossible to acquire from UPFs. Additionally, the development of food allergies depends on the combination of the dose, method of administration, and duration of exposure at an early time (36).

There are two main strengths of our study. The first is that all UPFs were identified from the individual's daily dietary intake rather than some specific components such as free fructose (20) and cookies (22). The second strength is that the data were based on an excellent, large, nationally representative sample to better present the associations between UPFs and allergic outcomes in this study. Due to the observational and cross-sectional nature of the NHANES database study, there are still several limitations. First, it was restricted to making causal inferences, as it is a cross-sectional study. Second, the reliability of such information based on self-reported questionnaire data is always an issue. Such questionnaires might be limited by recall bias, untruthful answers, etc. Third, IgE sensitization was simply defined as total IgE > 150 KU/L, as it was impossible to distinguish IgE levels caused by food allergy and/or other pathologies such as parasitic infection, auto-immune disorders, and neoplastic disease in samples of NHANES; it is needed to know that IgE sensitization has a certain expansion in this paper. Finally, UPFs were classified by NOVA, but the NHANES dietary survey was not specially designed to distinguish them according to NOVA. In the secondary classification, there is a certain misclassification bias that inevitably exists.

In conclusion, our study provided some evidence for the hypothesis that significant associations exist between UPFs and allergy symptoms in children and adolescents. As a considerable component of the modern and western diet, UPFs are new and important concepts based on the NOVA classification; awareness of its impact on allergy might help prompt the public about natural dietary patterns, which could improve the allergic immune defense in childhood.

## Data availability statement

Publicly available datasets were analyzed in this study. This data can be found here: https://www.cdc.gov/nchs/nhanes/search/default.aspx.

## Ethics statement

Detailed methods and protocols for the NHANES study were approved by the CDC/NCHS Research Ethics Review Board. They are publicly available through the CDC.gov website; this includes informed consent procedures for all participants. All methods in this study were performed according to the relevant guidelines and regulations. This study was exempt from human subject ethical review as the data are freely available in the public domain.

## Author contributions

WK conceived and designed the study. WK and YX extracted the data and analyzed and interpreted the data. WK and JZ completed the statistical analyzes and analyzed the data. WK and CC contributed to drafting and editing the paper and full access to all the data in the study and take responsibility for the integrity of the data and the accuracy of the data analysis. All authors have given the final approval of the manuscript.

## Conflict of interest

The authors declare that the research was conducted in the absence of any commercial or financial relationships that could be construed as a potential conflict of interest.

## Publisher's note

All claims expressed in this article are solely those of the authors and do not necessarily represent those of their affiliated organizations, or those of the publisher, the editors and the reviewers. Any product that may be evaluated in this article, or claim that may be made by its manufacturer, is not guaranteed or endorsed by the publisher.
